# High Frequency of Latent Conjunctival *C. trachomatis*, *M. hominis*, and *U. urealyticum* Infections in Young Adults with Dry Eye Disease

**DOI:** 10.1155/2014/154627

**Published:** 2014-05-22

**Authors:** Ernest V. Boiko, Alexei L. Pozniak, Dmitrii S. Maltsev, Alexei A. Suetov, Irina V. Nuralova

**Affiliations:** Department of Ophthalmology, Military Medical Academy, 5 Klinicheskaya Street, St. Petersburg 194044, Russia

## Abstract

*Aim*. To determine the frequency of detection of conjunctival *C. trachomatis* (CT), *M. hominis* (MH), and *U. urealyticum* (UU) infections in young adults with dry eye disease (DED), since these infections may potentially produce the chronic subclinical inflammation characteristic of DED. *Materials and Methods*. The study included subjects of 25–45 years of age, divided into the DED (*n* = 114) and nondry eye control (*n* = 98) groups, with the diagnosis based on self-reported complaints, biomicroscopy, the Schirmer I test, and break-up time. All patients had conjunctival scrapings taken to detect CT, MH, and UU with direct fluorescent-antibody assay kits. *Results*. At least one of the three microorganisms was found in 87.7% of the DED patients versus 8.2% of the controls. Of all the DED patients, 63.2%, 50.8%, and 42.1% were found to be infected with CT, MH, and UU, respectively. Multiple pathogens were identified in 65% of the DED patients found to be infected. CT infection was detected in 6.1% of the controls. *Conclusion*. *C. trachomatis*, *M. hominis*, and *U. urealyticum* were detected with high frequency in the conjunctiva of young adults with DED and may be an important risk factor for DED in them.

## 1. Introduction

The Dry Eye WorkShop (DEWS) definition of dry eye disease (DED) emphasizes the role of inflammation in the pathogenesis of this disease [1], which is reflected in the therapeutic strategies that have been used recently to treat DED [2, 3]. Some long-term clinical manifestations of inflammation, as conjunctival hyperemia, edema, and insignificant infiltration, are shared by both DED and chronic conjunctivitis. Between DED and chronic conjunctivitis, any significant diagnostic distinctions that can be revealed without special examination techniques are absent. Furthermore, the connections that have been revealed between some forms of conjunctivitis (in particular, the allergic one) [4] and DED indicate that chronic conjunctivitis may possibly result in DED. However, besides allergy, chronic inflammation of the conjunctiva also may be caused by persistent infection that, thus, leads to the development of DED.* C. trachomatis*,* M. hominis*, and* U. urealyticum* are the most common pathogenic microorganisms capable of persisting in tissues of human body for long time periods and causing not acute but mostly chronic low-grade nonspecific inflammation [5–7].

Because these infectious agents are those of sexually transmitted diseases, they are predominantly found in young adults [8–10]. Moreover, evidences of conjunctival localization with possible development of conjunctivitis have been reported for these pathogens and closely related species [11, 12].

Therefore, the aim of this study was to determine the frequency of detection of conjunctival *C. trachomatis*,* M. hominis*, and* U. urealyticum* infections in young adults with DED.

## 2. Materials and Methods

### 2.1. Design of the Study

This prospective case-control study was conducted at Military Medical Academy (St. Petersburg, Russia) during 2007 to 2012. The study adhered to the tenets of the Declaration of Helsinki and was approved by Ethics Committee of Military Medical Academy [13, 14].

### 2.2. Patients

The study included 212 subjects divided into two groups, the DED group (*n* = 114) and non-dry eye control group (*n* = 98). The inclusion criteria for DED group were age from 25 to 45 years, complaints of dryness, sensation of sand and/or foreign body sensation in the eye, insignificant conjunctival discharge and tearing (alone or in combinations), a Schirmer I test of 11 mm or less, and tear film break-up time (BUT) of 5 seconds or less. The nondry eye control group included nondry eye subjects of the same age range. Exclusion criteria included acute conjunctivitis, pathological lacrimal passages, contact lens wear, history of refractive surgery, and DED secondary to systemic diseases (Sjogren's syndrome, Reiter's syndrome, Stevens-Johnson syndrome, etc.), endocrine diseases, systemic diseases of connective tissue, current administration of antibiotic, anti-inflammatory, cytostatic, or hormonal agents, either locally or systematically, administration of oral contraceptives, and smoking.

### 2.3. Ophthalmic Examination

All patients underwent complete ophthalmic examinations and had conjunctival scrapings taken for direct fluorescent assay (DFA). Duration of the disease was self-reported by patients; Schirmer's I and BUT tests were performed to assess the severity of the disease. These are widely used and the most available dry eye diagnostic tests, with the sensitivity and specificity of the Schirmer I test reaching 85% and 100%, respectively, and those of the BUT test reaching 83% and 85%, respectively [1]. Because the DEWS recommends these two tests, along with clinical history, symptom questionnaires, and ocular surface staining grading, as those of the first five in “a practical sequence of tests” [1] for dry eye, they were used to detect DED in this study.

Schirmer's I test was performed by placing a Schirmer strip in the lateral lower conjunctival sac after instillation of one drop of topical 0.5% proxymetacaine (Alcaine, Alcon-Couvreur, Puurs, Belgium). Five minutes later, the amount of wetting was measured. To measure tear BUT, after instillation of a drop of sodium fluorescein dye (BioGlo Sterile Fluorescein Strips, HUB pharmaceutical, Rancho Cucamonga, CA), the tear film was observed under cobalt-blue filtered light of the slit lamp biomicroscope, and the interval between the last blink and appearance of the first break in the tear film was noted. Individual average BUT values were calculated from three repeated measurements.

### 2.4. Sampling

After instillation of one drop of topical 0.5% proxymetacaine (Alcon-Couvreur), each patient had conjunctival epithelial scraping taken from both eyes in a standardized manner, with the samples collected from tarsal conjunctiva and passed firmly four times across the conjunctiva. Then, the material obtained from a conjunctival scraping was spread on a slide and fixed in 70% cold methanol.

### 2.5. Direct Fluorescent Assay (DFA)

The method is based on binding of antibodies to an epitope (a specific trisaccharide component (aKdo-(2-8)-aKdo-(2-4)-aKdo) of cell wall lipopolysaccharide (LPS) for* C. trachomatis*, a surface protein antigen for* M. hominis*, or a surface protein antigen for* U. urealyticum*); currently, DFA tests are the only tests cleared by the Food and Drug Administration for the detection of ocular* C. trachomatis* infections [15]. Moreover, the DFA is of relatively low cost, easy, rapid, and suitable for routine use. The reported sensitivity and specificity of the DFA varies between 86% and 92% and 96% and 99% [16–18], respectively, in urogenital specimens, and approaches 100% and varies between 96% and 99%, respectively, in conjunctival scrapings [19]. The high rates of sensitivity and specificity of the DFA in the detection of ocular infection are attributed to the relative “purity” of conjunctival scrapings compared to urogenital specimens, and this is why the DFA actually conforms better to the detection of infection in the former than in the latter. For this reason, DFA method was chosen in this study.

**Figure 1:**
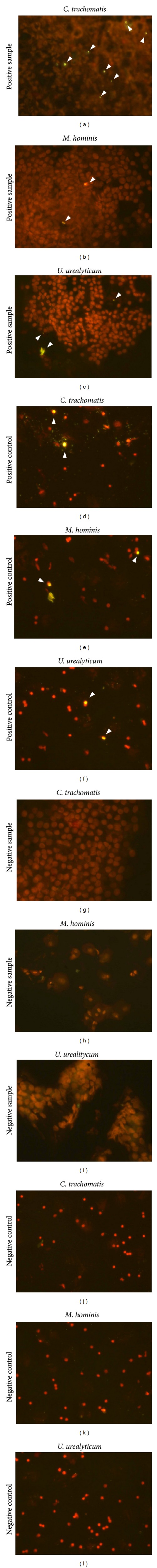
Direct fluorescence assay (DFA) staining for the detection of* C. trachomatis*,* M. hominis,* and* U. urealyticum* infection in conjunctival scrape smears of a dry eye patient ((a)–(c)), positive control slides ((d)–(f)), conjunctival scrape smears of a nondry eye patient ((g)–(i)), and negative control slides ((j)–(l)). Note the specific DFA staining patterns (small, well defined, round, apple-green or large, moderate bright green loci of fluorescence) in panels (a)–(f) (arrowheads) and absence of specific fluorescence in panels (g)–(l). DFA with Evans blue counterstain, original magnification ×400.

The polyclonal antibody based* C. trachomatis*,* M. hominis,* and* U. urealyticum* direct specimen kits, ChlamyScan, MicoScan, and UreaScan (LABDiagnostika, Moscow, Russia), respectively, were used for the detection of proper antigens according to the manufacturer's instructions. Briefly, conjunctival scrape smears were covered with 30 microliters of Evans blue counterstain containing solution of fluorescein-isothiocyanate- (FITC-) conjugated antibodies for 20 min at 20°C in a dark, humidified chamber. After being washed in phosphate buffer saline (PBS) and twice in distilled water, dried, and coverslipped with 10% glycerin solution in PBS, specimens were examined on Leica DM2500 microscope (Leica Microsystems GmbH, Wetzlar, Germany) (excitation wavelength, 490 nm; mean emission wavelength, 520 nm) equipped for FITC fluorescence. In* C. trachomatis* diagnostic tests, the positive-control was heteroploid line of L929 mouse fibroblasts (provided with ChlamyScan kit) infected with* C. trachomatis* strain L2 (Figure 1(d)). In* M. hominis* and* U. urealyticum* diagnostic tests, the positive-control (provided with MicroScan and UreaScan kits, resp.) contained suspension of HeLa cell culture separately infected with different strains of* M. hominis* and* U. urealyticum*, respectively (Figures 1(e), 1(f)). In* C. trachomatis*,* M. hominis,* and* U. urealyticum* diagnostic tests, the negative control contained conjunctival scrape smears of nondry eye patients and pathogens-free suspension culture of heteroploid L929 mouse fibroblasts (Figures 1(g)–1(l)). Evaluation was performed if the amount of epithelial cells in a scrape sample was at least 50. Loci of specific fluorescence were visualized at a magnification of ×400, with identification confirmed at a magnification of ×1000. The following was considered as a specific pattern: (1) small, well-defined, round, apple-green loci of fluorescence, located intracellularly or extracellularly or (2) large, moderate bright green loci of fluorescence, located intracellularly, corresponding to solitary cells and to intracellular inclusions of the pathogens, respectively (Figures 1(a)–1(c)). This pattern has been described as specific by the manufacturer and presented in some works [20, 21]. A sample was considered positive if at least 10 loci of specific fluorescence were identified, because this criterion has been found to provide an optimal ratio of sensitivity to specificity and used in a number of works [16–18]. If a uniocular infection was found, a patient was considered positive for infection.

### 2.6. Statistical Analysis

Nonparametric data analysis was performed with Statistica for Windows 6.0 software (Statsoft, Tulsa, OK). The Mann-Whitney *U* tests and Fisher's exact test were used to compare demographic parameters and results of the groups. Statistical significance was taken as *P* < 0.05.

## 3. Results

### 3.1. Characteristics of Patients and Results of Ophthalmic Examination

No statistically significant differences were noted between the DED and control groups in demographic characteristics (Table 1). In all patients of the DED group, consistent with DED symptoms (conjunctival hyperemia, complaints of dryness, smarting eyes, burning sensation, and foreign body sensation in the eye), Schirmer's I and BUT tests showed reduced tear production and destabilization of the tear film, respectively. In all patients of the control group, these characteristics were within normal ranges. These patients had neither complaints nor symptoms related to DED. In the DED group, the mean duration of the disease reported by 90.2% of the patients was 41.16 ± 9.12 months (range 37 to 58 months), with slow increase in the level of symptoms reported over time, whereas that reported by 9.8% of the patients was 22.92 ± 6.60 months (range 12 to 26.4 months).

### 3.2. DFA Results

**Figure 2:**
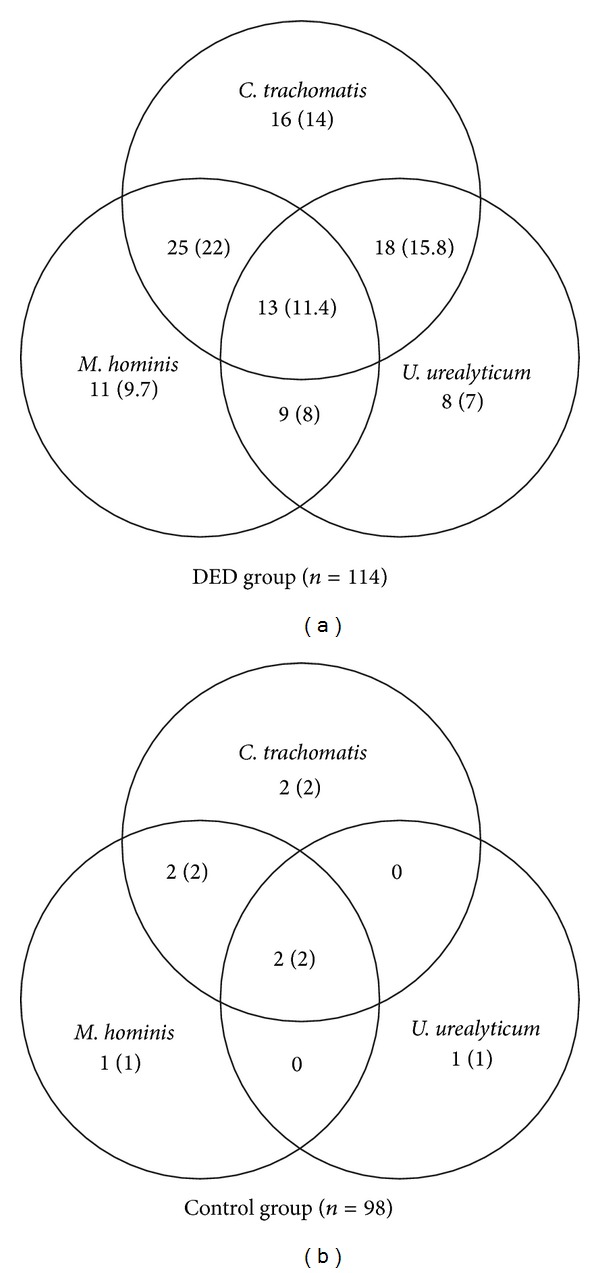
Distribution of* C. trachomatis*,* M. hominis*, and* U. urealyticum* infections in the DED group and nondry eye controls (*n* (%)). Statistical analysis showed a significant difference between the two groups with regard to the frequency of detection of (1) each of the three pathogens (*P* < 0.01) and (2) their mixed infections (*P* < 0.01).

At least one of the three microorganisms investigated in this study was found in 100 (87.7%) patients of the DED group versus 8 (8.2%) patients of the control group (Figure 2). Of all infected DED cases, only 35% were found infected with a single agent. Interestingly, of the DED patients infected with at least two pathogens, 86.2% were coinfected with* C. trachomatis*, which was found to be the most common infectious agent (72% in all infected study patients and 63.2% in the DED group). Besides,* C. trachomatis*, either alone or in association with other species, was identified in 8 (6.1%) patients of the nondry eye control group. During ophthalmic examination, no signs of chronic conjunctivitis or dry eye were found in the infected controls.

## 4. Discussion

This study showed that a large share of persons aged 25–45 years, with reduced tear production, destabilization of the tear film, conjunctival hyperemia, and complaints characteristic for DED, have chronic infectious conjunctivitis which might be caused by* C. trachomatis*,* M. hominis*, and* U. urealyticum* infections, either alone or mixed. This is in agreement with the statement that mild conjunctivitis is often associated with dry-eye patients [22] and suggests that, in persons of this age group, latent conjunctival infection is another important risk factor for DED.

The development of DED in young adults without any apparent risk factors for DED (age, history of refractive surgery, contact lens wear, systemic diseases or specific drug therapy, and obvious occupational risks) has no other possible explanation except for the action of a risk factor that has not yet been established (e.g., infectious agents). The complaints and clinical picture do not completely correspond to the conjunctival inflammation being characteristic for infectious damage, and this is the very reason why this chronic conjunctival infection is diagnosed as DED and not as conjunctivitis. Such cases of latent conjunctival infection might account for a part of the incidence of DED and require specific diagnostic and management approaches.

In this study, clinical manifestations of* C. trachomatis*-,* M. hominis*-, and* U. urealyticum*-induced chronic conjunctival inflammation were completely masked by DED symptoms and differed from manifestations of acute conjunctivitis (acute conjunctivitis was an exclusion criterion for enrollment). However, what needs to be explained is the fact that not all the patients found to be infected suffered from DED (in particular, infected controls had no manifestations of DED). Two of the most possible causes are (1) early stage of the disease and (2) genetically determined features accounting for intensity of the host conjunctival inflammatory response [23, 24]. Another possible cause is genetic variability in a pathogen, which is mostly a characteristic of* C. trachomatis* [23]. A limitation of the study is that serotyping was not performed. Thus, we do not know for sure whether association with DED cases is characteristic of the* C. trachomatis* species or of individual serovars within it [23]. In addition, other conjunctival bacterial microflora that might be of some potential value as a risk factor for DED was not investigated. Normal (saprophytic) conjunctival bacterial microflora may include a number of microorganism species that cause no inflammation and, therefore, are unlikely to have any value as a risk factor for DED. Most of the infectious agents being pathogenic for the conjunctiva, on the other hand, cause either acute or subacute conjunctivitis with a characteristic clinical picture that was not observed in DED patients of the study. Because these microorganism species were unlikely to play a role as a risk factor for DED in these patients, the control of the conjunctival bacterial microflora in them was not performed, and this could be considered a limitation of the study.

The inflammation associated with DED has the potential to promote conjunctival colonization, although predominantly by nonpathogenic and opportunistic microorganisms. The occurrence of rather contagious obligate pathogens such as* C. trachomatis* suggests that secondary colonization of already inflamed conjunctiva is not the case but indicates rather that these pathogens may play a primary role in the development and maintenance of inflammation; these issues, however, require further investigation. Association has already been established between DED and a number of infectious agents relating to such viral infections as human T-cell lymphotropic virus, human immunodeficiency virus, the Epstein-Barr virus, and hepatitis C virus [25]. These chronic viral infections trigger autoimmune reactions either initiating or contributing to lacrimal gland dysfunction in Sjogren's syndrome [25]. In those studies (reviewed by Alves et al. [25]), the subject of discussion has been autoimmune mechanisms and not the direct conjunctival or lacrimal gland damage induced by infectious agents. Yet, there is still a lot to be understood about the association between chronic conjunctival infections and non-Sjogren's dry eye, with the latter accounting for the major part of the incidence of DED [26]. Recently, the connection between DED and* Chlamydophila pneumoniae* infection in simultaneous clinical signs of follicular conjunctivitis has been reported, and, in that case, conjunctival localization of the agent as well as partial efficacy of etiotropic therapy has been proved [27]. Similar connection can be observed in infection with* C. trachomatis*, with the latter being a known cause of chronic conjunctival inflammation [28]. The role of* C. trachomatis* in the pathogenesis of DED may result from its high prevalence [9] and potential for persistence and support of chronic inflammation [5, 29]. These biological features of the infectious agent play a key role in the pathogenesis of endemic trachoma, which is caused by serovars A, B, Ba, and C only, whereas it is conjunctivitis that is caused by widespread serovars (D to K) of* C. trachomatis* [28]. In trachoma,* C. trachomatis*-induced conjunctival damage is characterized by marked alteration in the conjunctival tissue, lymphocytic infiltration, and scarring [24]. The same processes underlie the DED associated with infection, but in this case they are less active and result in either a gradual decrease in basal tear production or change in tear composition (due to the accompanying damage to accessory lacrimal glands and goblet cells). This explains why, in most (90.2%) of the patients, the mean duration of the disease was at least 3 years, with slow increase in the level of symptoms reported over time. And these are the long-duration cases of clinically asymptomatic disease showing no tendency to resolve spontaneously that are attributable to latent infection. Although persistence of the pathogen has been shown to be accompanied by changes in its morphology and epitope expression [30], this evidence is not used to confirm latent infection in clinical practice. The cellular morphology of the conjunctiva might also undergo changes during latent infection; assessing these changes was not the aim of the study.

Because localization of the infectious agent in only one of the two eyes of a patient is deemed unlikely, we did not study the association of unilaterally detected infection with manifestations of DED. Part of the reason for this unlikelihood is that interpretation of DFA is specific, with DFA positivity requiring detection of at least a cutoff number of loci of specific fluorescence and with ensuing false-positive results (e.g., those for a contralateral eye) [16].

The share of an infectious agent in general prevalence of DED may vary depending on the prevalence of this agent in the population. A rather high prevalence of ocular (conjunctival)* C. trachomatis* infection in persons aged 25–45 years may be related to the increased risk for urogenital infections for this age group [8–10]. Here, the infection can be transmitted to the conjunctiva by contact or hematogenously [20]. Our study provides evidence that* M. hominis* and* U. urealyticum* are two other infectious agents associated with chronic conjunctivitis and DED in persons aged 25–45 years. Although Mycoplasmataceae family members are also capable of damaging the conjunctiva, the clinical value of this fact has been unknown [12]. Because* M. hominis* and* U. urealyticum* also cause urogenital diseases and are of high prevalence in persons aged 25–45 years [7, 10], they might be one of the causes of low-grade conjunctival inflammation and DED in this age group. In this study, microbial coinfections were found more frequently (65%) than mono infections, which agrees with frequent detection of these coinfections in urogenital infections and supports the association of urogenital diseases with chronic conjunctivitis in patients of the age group. Moreover, because the association of chlamydial urogenital infection with chlamydial ocular diseases has been repeatedly reported [31], the association of urogenital diseases caused by these infectious agents with chronic conjunctivitis is also possible, but this issue has not been studied in this work and needs further investigation.

According to the International Dry Eye WorkShop, the disease comprises two subgroups: (1) evaporative dry eye and (2) aqueous-deficient dry eye; nevertheless, the etiopathogenetic subcategory of DED described in this study can be attributed to both of them [1]. Thus, this subcategory can belong to two DEWS classification categories, ocular surface disease (with the latter involving, e.g., allergy) and lacrimal deficiency (due to inflammatory infiltration and the ensuing reduction in basal tear production).

## 5. Conclusion

Latent* C. trachomatis*,* M. hominis*, and* U. urealyticum* infections are detected with high frequency in the conjunctiva of young adults with DED and may be an important risk factor for this disease in persons aged 25–45 years. This is associated with their potential for long-term damage to the conjunctiva and with high prevalence of these infectious agents among this age group. Therefore, it is deemed appropriate to conduct an examination for latent infections and, possibly, further antimicrobial treatment in some patients with DED.

## Figures and Tables

**Table 1:** Characteristics of patients in the dry eye disease group and control group.

Factor	*N*, total = 212		*P* value
	DED (*n* = 114)	Nondry eye controls (*n* = 98)	
Age in years, mean ± SD	35.6 ± 7.3	35.4 ± 7.1	0.51
Sex, male/female	49/65	48/50	0.08
Schirmer's *I* test, mm	7.9 ± 1.4	15.5 ± 0.6	<0.01
BUT, seconds	3.7 ± 0.6	11.4 ± 1.2	<0.01
